# Lactic acid bacteria alleviate neuronal excitotoxicity and extend *Caenorhabditis elegans* lifespan

**DOI:** 10.55730/1300-0152.2755

**Published:** 2025-06-23

**Authors:** Bao LE, Thi Nhat Hong NGUYEN, Seung-Hwan YANG

**Affiliations:** 1 Research Group in Pharmaceutical and Biomedical Sciences, Faculty of Pharmacy, Ton Duc Thang University, Ho Chi Minh City, Vietnam; 2 Department of Biotechnology, Chonnam National University, Yeosu, Chonnam, Republic of Korea

**Keywords:** Antioxidant, *Caenorhabditis elegans*, lactic acid bacteria, neurological disorders, paraprobiotic

## Abstract

**Aim:**

Neuronal cell death plays a critical role in the development of neurological disorders associated with aging. This study aimed to evaluate the beneficial effects of heat-ki lled lactic acid bacteria (hkLAB) on neuroblastoma cells and *Caenorhabditis elegans*.

**Materials and methods:**

We pretreated heat-killed *Lactobacillus fermentum* CNU384 (hkCNU384), *L*. *brevis* CNU386 (hkCNU386), *L*. *helveticus* CNU395 (hkCNU395), and *L*. *paracasei* CNU396 (hkCNU396) at a concentration of 10^9^ CFU/mL to investigate their neuroprotective effects on glutamate-induced SH-SY5Y cells and their impact on the lifespan of 6-hydroxydopamine (6-OHDA)-induced *C*. *elegans*.

**Results:**

Our findings indicate that hkCNU395 and hkCNU396 protected against glutamate-induced cell death via modulation of the Bax/Bcl-2 apoptosis regulator ratio and enhancement of endogenous antioxidant enzyme activity. Moreover, hkCNU396 significantly improves survival rates in *C*. *elegans* with neuron degeneration induced by 6-OHDA.

**Conclusion:**

These results highlight hkCNU396 as a promising paraprobiotic candidate for patients suffering from degenerative neurodegenerative diseases.

## 1. Introduction

Despite the availability of many approved therapies of neurodegenerative disorders (NDs), most treatments only control the symptoms and fail to control disease progression. Furthermore, patients have to cope with numerous side effects of available treatments (Gadhave et al., 2024). It is important to understand the precise mechanisms of NDs to identify new therapeutic targets and develop more effective therapies. However, a significant knowledge gap persists regarding the interaction of multiple crucial factors such as genetic alteration, oxidative damage, neuroinflammation, and mitochondrial dysfunction in NDs (Yu et al., 2024). Maintaining oxidative homeostasis is crucial for the proper functioning of nerve cells. Oxidative stress leads to damaged cellular macromolecules (DNA and proteins) and triggers lipid peroxidation, ultimately hastening tissue death and cellular apoptosis (Houldsworth, 2024).

Current advanced therapies such as gene therapy, immunotherapy, and biomaterials for delivering drugs effectively raise safety and efficacy concerns (Kumbhar et al., 2024; Singh and Nagdev, 2024). The concept of using the gut microbiome as a therapeutic tool to regulate brain function has emerged as a potential therapeutic approach, rooted in the effects of essential metabolites (Mohajeri et al., 2018). Changes in gut microbiota occur dynamically over time, impacted by factors including age, psychological health, lifestyle patterns, and digestive system status. The gut microbiota actively shapes host pathophysiology, highlighting its importance for clinical diagnostics and therapy (Olsson et al., 2022). The supplementation of probiotics or paraprobiotic (inactivated probiotics) may promote the growth of particular microbes for short or long durations, modify the characteristics and functions of the microbiome and establishing interactions with the host (Barros et al., 2020). Research has shown that probiotics can help mitigate or prevent various diverse illnesses, including neurological disorders. Oral administration of *L*. *plantarum* DP189 (DP189) has been reported to suppress the pathological states of Parkinson’s disease (PD) in mice by delaying the accumulation of αSyn, inhibiting oxidative stress, inhibiting the proinflammatory response, and reshaping the gut microbiota (Wang et al., 2021, 2022). Tamtaji et al. (2019) carried out a 12-week randomized, double-blind, placebo-controlled trial using a probiotic mixture (including *L*. *acidophilus*, *Bifidobacterium bifidum*, *L*. *reuteri*, and *L*. *fermentum*) in patients with PD. Patients showed improvements in inflammation, oxidative stress, and insulin metabolism biomarkers. Despite promising outcomes of microbial-targeted therapy, clinical trials have failed to achieve desired therapeutic outcomes. Hence, there is a pressing need to focus on prioritizing novel strains, identifying key metabolites, and regulating gastrointestinal microbiota for effective disease prevention and management.

Paraprobiotics are nonviable microbial cells that offer enhanced safety for immune-compromised individuals, improved stability under extreme conditions, and no risk of antibiotic resistance. Human neuroblastoma SH-SY5Y cells are the most widely used in vitro cell model for neurotoxicity due to their immortalized nature and ease of culture (Sun et al., 2010; Lee et al., 2019; Nigro et al., 2023). Herein, we sought to evaluate the protective effects of heat-killed probiotics against glutamate-induced neuronal cells and assessed the underlying mechanisms involved in cell deaths via apoptosis and oxidants stress. *Caenorhabditis elegans* is a short-lived, nonparasitic nematode with over 80% of its genome homologous to humans, making it a valuable model for studying aging and neurodegenerative diseases (Prahlad and Morimoto, 2009; Li and Le, 2013). We investigated the effects of heat-killed lactic acid bacteria (hkLAB) on the lifespan extension of *C*. *elegans*.

## 2. Materials and methods

### 2.1. C. elegans, SH-SY5Y cell culture, and bacterial growth conditions

Bacterial strains *L*. *fermentum* CNU384, *L*. *brevis* CNU386, *L*. *helveticus* CNU395, and *L*. *paracasei* CNU396, obtained from the Yang Lab Culture Collection at Chonnam National University, Republic of Korea, were prepared by enrichment culturing in de Man, Rogosa, and Sharpe (MRS) medium. The cultures were incubated at 37 °C under anaerobic conditions with shaking for 24 h. Whole bacterial cells were collected, rinsed with phosphate buffered saline (PBS), and centrifuged. The pelleted cells were gently resuspended in PBS through vortexing and subjected to heat treatment at 100 °C for 10 min to obtain heat-killed *L*. *fermentum* CNU384 (hkCNU384), *L*. *brevis* CNU386 (hkCNU386), *L*. *helveticus* CNU395 (hkCNU395), and *L*. *paracasei* CNU396 (hkCNU396). The human neuroblastoma SH-SY5Y cell line was purchased from the Korean Cell Line Bank (Republic of Korea) and were grown in DMEM enriched with 4.5 g/L glucose, 10% fetal bovine serum (FBS), and penicillin-streptomycin (100 U/mL) (Capricorn Scientific, Ebsdorfergrund, Germany) at 37 °C in 5% CO_2_. Experiments were conducted 24 h after seeding the cells onto plates. Wild-type N2 *C*. *elegans* were provided by the Caenorhabditis Genetics Center (CGC, University of Minnesota, Minneapolis, MN, USA). Nematodes were maintained on Nematode Growth Media (NGM) agar plates at 20 °C coated with live *Escherichia coli* bacteria (OP50).

### 2.2. Cell viability assay

The assay procedure was performed based on Lee et al. (2019) with minor modifications. SH-SY5Y cells were differentiated by treatment with 10 μM transretinoic acid, with the medium replaced every 2 days using fresh RA (10 μM) and 1% FBS for 7 days. Differentiated SH-SY5Y cells were seeded at a final concentration of 1 × 10^5^ cells/well in a 96-well plate. After 24 h, the cells were treated with 10^9^ CFU/mL of the heat-killed bacterial strains (hkCNU384, hkCNU386, hkCNU395, and hkCNU396) for an additional 2 h, and subsequently exposed to glutamate (100 mM) or a control condition for 24 h. After incubation, the plate was rinsed with PBS, followed by the addition of 10 μL of MTT reagent to each well. The absorbance (OD) was measured at 450 nm using a microplate reader after a 4 h incubation period. Cell viability was calculated relative to the negative control (NC) group, which was not treated with heat-killed bacterial strains.

### 2.3. Western blot

Western blots of relevant proteins in SH-SY5Y cells were performed by running the cell lysates on SDS-PAGE and subsequently immunoblotting the following antibodies: Bax (1:1000, catalog number 20067), Bcl-2 (1:1000, catalog number 7382), SOD1 (1:1000, catalog number sc-515404), CAT (1:1000, catalog number sc-271358), GR (1:1000, catalog number 133245), and β-actin (1:2000, catalog number 58673) (all from Santa Cruz Biotechnology, Santa Cruz, CA, USA). After 3 washes with PBS-T for 10 min each, the membranes were incubated at room temperature for 1 h with mouse IgG Fc binding protein (m-IgG Fc BP) conjugated to horseradish peroxidase (HRP) (1:1000, catalog number 525409, Santa Cruz Biotechnology, Santa Cruz, CA, USA). Proteins were detected using chemiluminescence (ATTO, Tokyo, Japan). Densitometry of the protein bands was analyzed using ImageJ software.

### 2.4. qRT-PCR

Total RNA was isolated using the Qiagen RNeasy Mini kits (Qiagen, Hilden, Germany). Subsequently, cDNA was synthesized using PrimeScript RT Master Mix (Takara Korea Biomedical Inc., Seoul, Republic of Korea) according to the manufacturer’s instructions. The qPCR reaction was performed using SYBR Premix Ex Taq (Takara Korea Biomedical Inc., Seoul, Republic of Korea), following the manufacturer’s instructions. Normalized expression changes were calculated using the comparative quantification method 2^−ΔΔCt^ (with the control *GAPDH* set to 1). Table lists the primer pair sequences used to amplify *Bax*, *Bcl-2*, superoxide dismutase (*SOD*), catalase (*CAT*), glutathione reductase (*GR*), and *GAPDH* (used as the internal reference gene). The PCR conditions were as follows: 95 °C for 2 min, followed by 40 cycles at 95 °C for 1 min, and 60 °C for 20 s.

### 2.5. Lifespan assay

For lifespan analysis, wild-type N2 nematodes were cultured on solid NGM plates with 50 mM 6-OHDA and either a 10^9^ CFU/mL concentration of heat-killed strains (hkCNU384, hkCNU386, hkCNU395, hkCNU396) or the control *E*. *coli* OP50, with this setup designated as day 0. Approximately 40 nematodes were used for each replicate and transferred to new plates every other day. Nematodes unresponsive to gentle mechanical stimulation at the head using a picker were recorded as dead. Worms showing bagging, explosion, or leaving the plates were excluded from the count. Each lifespan experiment included 3 biological replicates.

### 2.6. Statistical analysis

All statistical analyses were conducted using one-way analysis of variance (ANOVA), followed by Dunnett’s honestly significant difference (HSD) test in GraphPad Prism 9 and SPSS 21.0. Results are presented as the mean ± standard error of the mean (SEM), with a p-value of less than 0.05 considered statistically significant.

## 3. Results and discussion

### 3.1. hkLAB suppresses glutamate-induced cytotoxicity in SH-SY5Y cells

**Figure 1:**
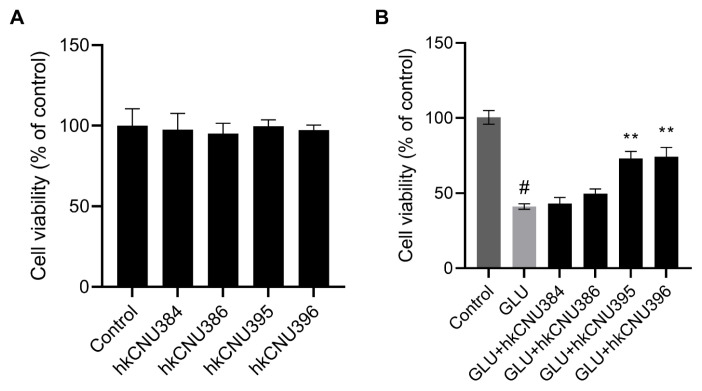
Effect of hkLAB on the viability of SH-SY5Y cells. SH-SY5Y cells were treated with hkLAB (10^9^ cells/mL) alone for 24 h (A). SH-SY5Y cells were pretreated with hkLAB (10^9^ cells/mL) for 2 h, followed by treatment with glutamate (100 mM) for another 24 h (B). Data are expressed as the mean ± SD (n = 3). #p < 0.05 vs. control group, **p < 0.01 vs. glutamate (100 mM, GLU) treated group.

Glutamate excitotoxicity induces neuronal death through the dysfunction of mitochondrial fission processes in the mammalian central nervous system (CNS) (Michaelis, 1998). Glutamate-induced excitotoxicity has been associated with oxidative stress and has emerged as a potential therapeutic target for treating NDs (Atlante et al., 2001). In the present study, we confirmed that glutamate is highly toxic to SH-SY5Y cells. To evaluate the effects of heat-killed bacterial strains on glutamate-induced cell death, we first determined the toxicity of hkLAB (10^9^ CFU/mL) on SH-SY5Y cells (Figure 1A). Cell viability did not differ significantly in hkLAB-treated SH-SY5Y cells, confirming the safety of hkLAB at the concentrations used in this study. As shown in Figure 1B, the viability of SH-SY5Y cells treated with glutamate (100 mM) alone significantly decreased to 41.1 ± 1.80% compared with control cells (100%, p < 0.05). However, pretreatment with hkLAB for 2 h showed protective effects. Heat-killed *L*. *helveticus* CNU395 and *L*. *paracasei* CNU396 increased the viability of treated cells to 73.1 ± 4.76% and 74.2 ± 6.16%, respectively (p < 0.05). In contrast, treatment with heat-killed *L*. *fermentum* CNU384 and *L*. *brevis* CNU386 did not enhance cell viability.

### 3.2. hkLAB protected against glutamate-induced apoptosis

**Figure 2:**
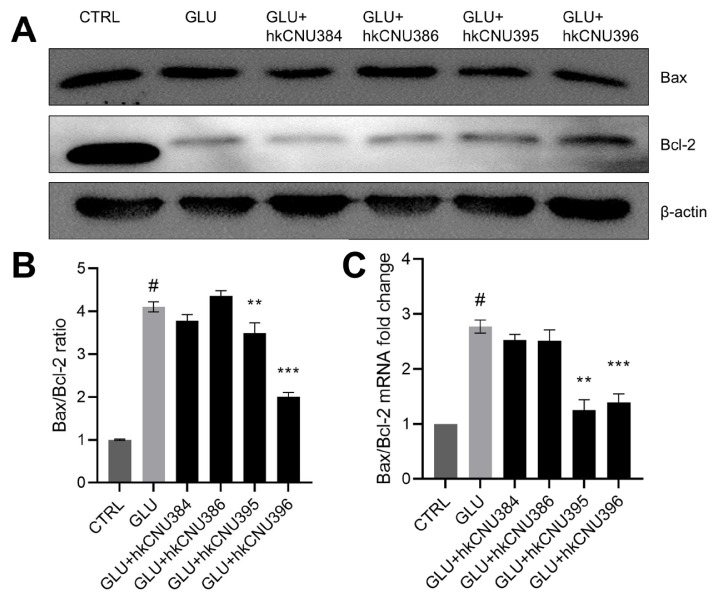
Effect of hkLAB on the expression of Bax and Bcl-2 in SH-SY5Y cells. (A) The levels of Bax and Bcl-2 proteins were measured by western blot. (B) Quantitative analysis of the protein bands of the Bax and Bcl-2. (C) mRNA expression levels of Bax and Bcl-2 were analyzed by RT-qPCR. The data shown is representative of 3 separate experiments with similar results. Data are expressed as the mean ± SD (n = 3). #p < 0.05 vs. control (CTRL) group, **p < 0.01, ***p < 0.001 vs. glutamate (100 mM, GLU) treated group.

In programmed cell death (apoptosis), Bax promotes mitochondrial membrane permeabilization, while Bcl-2 counteracts this process to maintain cellular survival (Qian et al., 2022). Increased Bax expression and decreased Bcl-2 levels initiate a cascade of events driving cellular apoptosis. Previous studies have shown that glutamate triggers an imbalance in the Bax to Bcl-2 ratio, contributing to the progression of several NDs (Kumar et al., 2010). This prompted us to investigate whether hkLAB can protect against glutamate-induced excitotoxic neurodegeneration. To test this hypothesis, SH-SY5Y cells were treated with 100 mM glutamate for 24 h (Figures 2A and 2B). Glutamate exposure caused a significant 4-fold increase in the Bax/Bcl-2 ratio (p < 0.05). Pretreatment with hkCNU395 and hkCNU396 prior to glutamate exposure reduced Bax/Bcl-2 expression and restored the balance between Bax and Bcl-2 by 3.24-fold and 1.93-fold, respectively. However, treatments with hkCNU384 and hkCNU386 showed no significant change in Bax/Bcl-2 expression. For further confirmation, gene expression analysis in SH-SY5Y cells showed that hkCNU395 and hkCNU396 treatments significantly decreased the Bax/Bcl-2 ratio (Figure 2C), consistent with observations of protein expression. These results suggest that *L*. *helveticus* CNU395 and *L*. *paracasei* CNU396 protect SH-SY5Y cells against glutamate-induced cell damage by regulating the apoptosis pathway.

### 3.3. hkLAB increased the expression of antioxidant enzymes in glutamate-induced SH-SY5Y cells

**Figure 3:**
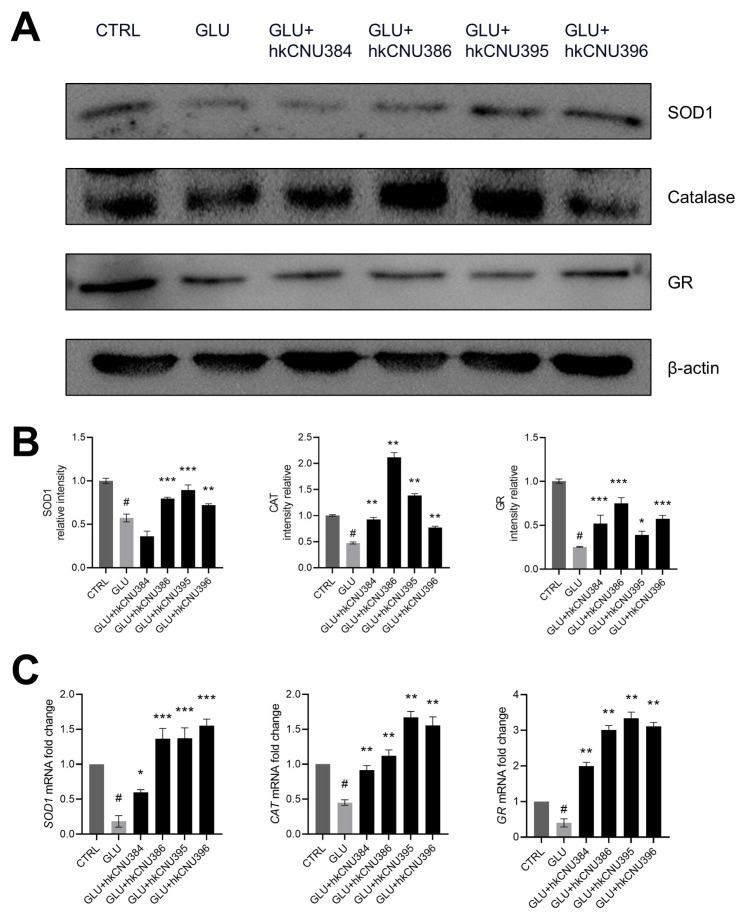
Effect of hkLAB on the expression of antioxidant enzymes in SH-SY5Y cells. (A) The levels of SOD1 (superoxide dismutase), CAT (catalase), and GR (glutathione reductase) enzymes were measured by western blot. (B) Quantitative analysis of the protein bands of the SOD1, CAT, and GR. (C) mRNA expression levels of *SOD1*, *CAT*, and *GR* were analyzed by RT-qPCR. The data shown is representative of 3 separate experiments with similar results. Data are expressed as the mean ± SD (n = 3). #p < 0.05 vs. control (CTRL) group, *p < 0.05, **p < 0.01, ***p < 0.001 vs. glutamate (100 mM, GLU) treated group.

To elucidate the potential mechanism by which hkLAB enhance survival rates of SH-SY5Y, we analyzed protein expression of antioxidant enzymes. SOD plays a critical role in neutralizing superoxide ions by converting them into molecular oxygen and hydrogen peroxide, thus mitigating oxidative stress (Chen et al., 2024). CAT further decomposes hydrogen peroxide into harmless water and oxygen, preventing cellular damage caused by excess reactive oxygen species (Houdou et al., 1991). Additionally, GR is essential for maintaining adequate levels of reduced glutathione, a crucial molecule for preserving cellular redox balance and protecting cells from oxidative injury (Dringen, 2000). Glutamate-induced SH-SY5Y cells had a downregulation of SOD1, CAT, and GR, confirming the oxidative toxicity of glutamate in SH-SY5Y cells after 24 h of exposure (Figure 3). However, this effect was improved in cells pretreated with hkLAB. Interestingly, the expression of SOD1 was not significantly different in cells pretreated with hkCNU384 compared to those treated with the control, suggesting that only certain strains can regulate expression of antioxidant enzymes. To further confirm the expression of antioxidant enzymes, we detected higher levels of SOD1, CAT, and GR in glutamate-induced cells than control cells. Antioxidant enzymes, such as SOD1, CAT, and GR, play important roles in maintaining an appropriate intracellular oxidation status. Antioxidant enzyme level decreases are associated with NDs as well as brain and spinal cord damage. Thus, pretreatment with hkLAB notably suppresses oxidative stress-induced cell death by upregulating oxidant enzymes in maintaining cellular oxidative balance.

### 3.4. hkLAB prolongs lifespan of C. elegans

**Figure 4:**
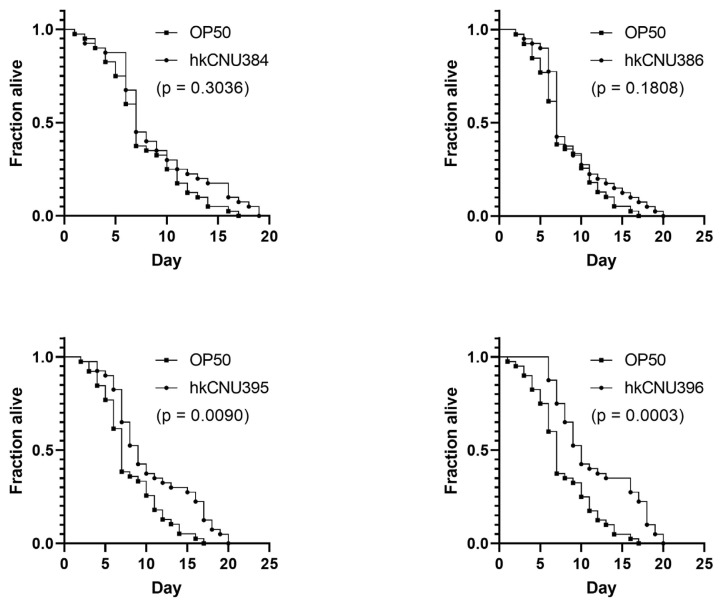
Effect of hkLAB on the N2 *C*. *elegans*. Data are expressed as the mean ± SD (n = 3).

Emerging evidence indicates that 6-OHDA is a common toxin that can be used to induce neurodegeneration in animal models (Schober, 2004). We next evaluated the role of hkLAB in lifespan of 6-OHDA-induced *C*. *elegans* in vivo. To examine the impact of hkLAB on lifespan, we used hkLAB as an alternative diet to *E*. *coli* OP50 (the control diet) of wild-type N2 worms. Previous studies have shown that the administration of *Agathobaculum butyriciproducens* SR79^T^ had protective effects against 6-OHDA-induced toxicity in mouse models regulating the AKT/Nrf2/ARE signaling pathway and astrocyte activation (Ryu et al., 2022). Another study by Francisco and Grau (2025) reported that biofilm-forming *Bacillus subtilis* promoted the lifespan extension in a *C*. *elegans* model by targeting dopaminergic neurons and improving dopaminergic-dependent behaviors. Here, we observed a higher survival rate in 6-OHDA-induced *C*. *elegans* treated with hkCNU396 compared to control group, suggesting hkCNU396 can enhance lifespan (Figure 4).

## 4. Conclusion

In this study, we explored the endogenous cellular and molecular mechanisms underlying the neuroprotective effects of hkLAB against glutamate excitotoxicity, protecting neural cells from oxidant-mediated glutamate toxicity. We unexpectedly discovered that the effects of hkLAB vary depending on the bacterial strain. Interestingly, hkLAB was found to protect against glutamate-induced apoptosis in neuroblastoma cells by regulating the Bax to Bcl-2 ratio and increasing the expression of antioxidant enzymes. Furthermore, only hkCNU396 enhanced the lifespan of *C*. *elegans* in an in vivo study. In summary, our work identifies heat-killed *L*. *paracasei* CNU396 as a promising paraprobiotic candidate for patients with degenerative neurodegenerative diseases. However, as numerous intrinsic and environmental factors influence the interaction between gut microbes and the host, further studies are required to fully understand its effectiveness and regulatory mechanisms.

## Figures and Tables

**Table:** List of primers used in qPCR.

Gene primer	Accession number	Sequence
Bax	NM_001291428	GGCTGTCCTGGAACTGGCTTTG
		TCTCTCTCCATGCCCTCTGT
Bcl-2	NM_000633	GGATGCCTTTGTGGAACTGT
		AGCCTGCAGCTTTGTTTCAT
Superoxide dismutase (SOD1)	NM_000454	AGGGCATCATCAATTTCGAG
		ACATTGCCCAAGTCTCCAAC
Catalase (CAT)	NM_001752 XM_014729341	ACCAAGGTTTGGCCTCACAA
		TTGGGTCAAAGGCCAACTGT
Glutathione reductase (GR)	NM_000637	AGCTTAGCGTTCATCCGTGT
		TCCAATCATCCGTCAAAACA
Glyceraldehyde-3-phosphate dehydrogenase (GAPDH)	NM_001357943	ACAGTCAGCCGCATCTTCTT
		GACAAGCTTCCCGTTCTCAG
